# Book Review: Safer Healthcare; Strategies for the Real World

**DOI:** 10.3389/fpubh.2017.00131

**Published:** 2017-06-06

**Authors:** Gavan Lintern

**Affiliations:** ^1^Monash University Accident Research Centre, Monash University, Melbourne, VIC, Australia

**Keywords:** safety management, healthcare, safety models, healthcare safety, systems safety

It has been a decade or more since anything in safety management has grabbed my attention. We seem to be hashing over the same ideas and not making much progress. Problematically, the field is littered with diverse ideas that assemble into a poorly integrated and inconsistent whole. Worse, implementation of some of the more compelling ideas will arguably increase risk of harm. With these thoughts in mind, I was poised to dismiss this book by Vincent and Amalberti as more of the same. I turned around on that rather quickly. I was barely into Chapter 1 before I encountered a fresh way of conceptualizing the healthcare safety management problem. More generally, there are ideas in this book that can help us reformulate our approach to safety management not only in healthcare but also in other industries. Because there are far too many fresh insights to cover in this review, I will constrain myself to covering those that jumped out at me.

Vincent and Amalberti argue that different industrial settings require different approaches to safety. For example, if deep-sea fishing captains operated under the procedural constraints imposed on commercial aviation, there would be no deep-sea fishing industry. Commercial aviation operates under an ultra-safe model in which risk is controlled or avoided where possible, while deep-sea fishing operates under an adaptive model in which operators accept risk and rely on their expertise to cope with hazardous conditions.

As an industry, healthcare is unusual in that it encompasses a wide range of industrial models. Some areas of healthcare are amenable to managing risk by use of procedures and standards because they can control demand, work flow, and working conditions. Radiotherapy, for example, is highly regulated by a web of procedures and standards that would be entirely disruptive if they were imposed on emergency surgery which, necessarily, relies on an adaptive approach to safety.

This view that there is a range of safety models and that the particular safety model used in any industrial environment must conform to the pragmatic constraints on the work is a valuable insight. Scholarly treatments typically frame safety management in terms of a single model, with the implication that it is the model we should follow in all settings. Within healthcare at least, a commitment to one universal safety model is clearly inadequate.

Vincent and Amalberti also observe that healthcare safety has focused almost exclusively on singular events at point-of-care within hospital settings (e.g., central line infections). They argue that patient welfare involves an accumulation and progression through the healthcare system, where isolated errors and incidents are generally less important than overall coordination of care. Thus, we need a broader view of safety management. Along with safety interventions at point-of-care (e.g., standardization and enhancement of handover) we need organizational interventions (e.g., staffing-level management) and system interventions (e.g., guidelines for management of patients with complex conditions).

Furthermore, developments in healthcare, particularly with respect to invasiveness of surgical treatments, are reducing the time patients spend in hospital. As populations age and as technological developments further reduce the invasiveness of serious treatments, we can expect the balance to swing further away from hospital, thereby accentuating the need to be concerned with healthcare safety in primary care and in the home. For this brief review, three issues are illustrative.

Once a patient is released from hospital, coordination between the different healthcare practitioners who remain on the case becomes more difficult. However, the patient, who necessarily participates in all care events, develops a unique vantage point. They (or a family member) could be viewed as care coordination resource, thereby allowing them to take responsibility for one important safety-related care function. Healthcare professionals may need to acknowledge, promote, and respect that patient role formally and explicitly. Further, to make this work systemically, the patient (or family member) may need some form of training in care coordination.

In addition, post-hospitalization care invariably requires the patient or a family member to perform essential healthcare tasks. We need to consider whether we can relax safety standards in transition from hospital to home; for example, should we demand the same level of sterility in the home that is mandatory for dressing changes in a hospital? Alternatively, can we allow pragmatic relaxation of safety standards when a patient moves from hospital to home?

Finally, hospitals are well placed to respond rapidly to health crises experienced by their patients. This is a significant vulnerability in home healthcare and may constitute a prime target for development of organizational interventions that facilitate rapid response to health crises in the home.

To summarize, we have been flailing around in healthcare safety management, looking for a way forward. I doubt this book offers the definitive answer, but it does offer a suite of ideas that can get us heading in a productive direction. Possibly, its most valuable aspect is that it offers a comprehensive structure as a way of thinking about safety management in this most complex and diverse of endeavors. And if you are concerned with safety management in another industry, this book will offer good value. Other industries may not have the functional diversity of healthcare, but the structure offered here will help you sift out from all that is written about safety management what is relevant to your industry from what is not.

## Author Contributions

The author confirms being the sole contributor of this work and has approved it for publication.

## Conflict of Interest Statement

The author declares that the research was conducted in the absence of any commercial or financial relationships that could be construed as a potential conflict of interest.

